# How promising is phototherapy for cancer?

**DOI:** 10.1038/s41416-020-0926-3

**Published:** 2020-06-26

**Authors:** Huayun Shi, Peter J. Sadler

**Affiliations:** Department of Chemistry, University of Warwick, Coventry, CV4 7AL UK

**Keywords:** Pharmaceutics, Medicinal chemistry

## Abstract

Oncological phototherapy, including current photodynamic therapy (PDT), developmental photoactivated chemotherapy (PACT) and photothermal therapy (PTT), shows promising photo-efficacy for superficial and internal tumours. The dual application of light and photochemotherapeutic agents allows accurate cancer targeting, low invasiveness and novel mechanisms of action. Current advances in new light sources and photoactive agents are encouraging for future development.

## Main

Light was first applied 4000 years ago in ancient Egypt to treat vitiligo, but the modern clinical usage of anticancer photodynamic therapy (PDT) began at the Roswell Park Cancer Institute in the 1970s. Efficacy switched-on by irradiation makes the treatment highly controllable, spatially and temporally with minimal invasiveness. In current clinical PDT, photosensitisers excited by light generate cytotoxic radicals by interaction with biomolecules (Type I pathway), or, in the Type II pathway, convert ground-state oxygen (^3^O_2_) directly into highly reactive cytotoxic excited-state singlet oxygen (^1^O_2_).^1^ PDT can not only kill cancer cells directly, but also cause vascular damage that impedes tumour oxygen supply.

**Fig. 1:**
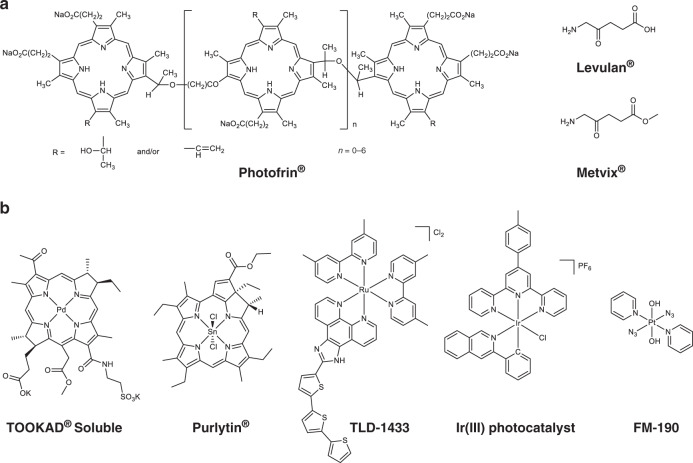
**a** FDA-approved anticancer photosensitisers; **b** metal-based photoactive anticancer complexes under development.

Photosensitisers are key components of PDT (Fig. 1, Table 1). An ideal photosensitiser shows low dark cytotoxicity, tumour-specific accumulation, high photocytotoxicity, low skin photosensitivity and facile administration.^1,2^ The first-generation photosensitiser Photofrin^®^, a mixture of oligomeric haematoporphyrin derivatives (HpD), was approved to treat bladder cancer with red light (630 nm) in 1993 in Canada, and remains the most widely used photosensitiser, despite its poor cancer selectivity, low red-light absorption and long-term photosensitivity. 5-Aminolevulinic acid (Levulan^®^), a key precursor for biosynthesis of protoporphyrin IX, and its derivative methyl aminolevulinate (Metvix^®^), are readily synthesised in high purity, and approved by the FDA as second-generation photosensitisers. Peripheral double backbone reduction of porphyrin photosensitisers can red-shift and strengthen their longest-wavelength absorbance. Thus, chlorin derivatives Foscan^®^, Radachlorin^®^ and Laserphyrin^®^ have been approved for clinical PDT with red light (ca. 660 nm), and bacteriochlorin derivative Redaporfin, in clinical Phase 2, can be activated with infrared light (749 nm).

**Table 1:** PDT agents in clinical use or in clinical trials^a^.

Class	PDT agent	Metal	Stage	Excitation (nm)	Area	Cancer type
Protoporphyrin IX precursor	5-Aminolevulinic acid (Levulan^®^)		FDA approved	635	Global	Skin, brain, oesophagus
	Methyl aminolevulinate (Metvix^®^)		FDA approved	635		Skin
	Hexyl 5-aminolevulinate (Hexvix^®^)		FDA approved	380–450 (diagnosis)		Bladder
Porphyrin derivatives	Porfimer sodium (Photofrin^®^)		FDA approved	630	Global	Lung, bladder, oesophagus, bile duct, brain
	Photogem		MHRF approved	660	Russia	Respiratory and digestive tracts, urogenital
Chlorin derivatives	Temoporfin (Foscan^®^)		EMA approved	652	EU	Head and neck, bile duct, lung
	Ce6-PVP (Fotolon^®^)		Phase 2	660–670	Germany	Lung
	Radachlorin^®^		MHRF approved	662	Russia	Skin
	Talaporfin sodium (Laserphyrin^®^)		MHLW approved	664	Japan	Lung, brain
	HPPH (Photochlor^®^)		Phase 2	665	USA	Lung, oral cavity, oesophagus
Bacteriochlorin derivatives	Redaporfin		Phase 2	749	Portugal	Head and neck
Phthalocyanine derivatives	Silicon phthalocyanine (Pc4)		Phase 1	672	USA	Skin
Metal complex	Padoporfin (TOOKAD^®^)	Pd	Terminated	763	EU	Prostate
	Padeliporfin potassium (TOOKAD^®^ Soluble)	Pd	EMA approved	753	EU	Prostate
	TLD-1433	Ru	Phase 2	520	Canada	Bladder, brain
	Motexafin lutetium (Antrin^®^)	Lu	Terminated	732	USA	Breast, prostate
	Rostaporfin (Purlytin^®^)	Sn	Phase 2/3	664	USA	Breast, bile duct, ovarian, colon

^a^Data from clinicaltrials.gov.

Metal complexes offer potential advantages in the design of photosensitisers, since the metal can improve stability and photocytotoxicity, and allow their quantification and localisation by  for exmaple ICP-MS.^2^ Also, with the help of advanced mass spectrometry, the molecular effects of metal-based photosensitisers on biochemical pathways in cancer cells can be probed effectively.^2^ Vascular-targeted Tookad^®^ Soluble is a Pd(II)-bacteriopheophorbide approved in the EU to treat localised prostate cancer with near-infrared (NIR) irradiation (753 nm). A Sn(IV)-chlorin derivative Purlytin is in Phase 2/3 trials for cutaneous metastatic breast cancer, and TLD-1433, a polypyridyl Ru(II) complex, has entered clinical trials for non-muscle-invasive bladder cancer (NMIBC).^3^

The excitation wavelengths of photosensitisers are restricted by their absorption spectra and affect light tissue penetration that varies in different organs. Usually, PDT is performed at wavelengths >620 nm, since longer wavelength light penetrates more deeply.^3,4^ However, for superficial tumours (e.g. NMIBC), green light may be more effective and able to circumvent unwanted side effects on healthy tissue. In addition, pain and discomfort caused by red-light PDT can be significantly reduced using daylight PDT to treat precancerous skin lesions.^5^ Daylight skin PDT can be performed using sunlight (>4–8 J/cm^2^), but light devices are necessary for internal tumours, and for skin cancer in poor sunlight.^4^ Lasers as PDT light sources offer high power output and easy connection to optical fibres and endoscopes, with precise light delivery to tumours in internal organs. However, due to the narrow spectral width, small beam cross-section, cost and handling requirements of lasers, broadband lamps with specific filters are usually preferred to treat large superficial lesions directly (e.g. non-melanoma skin cancer). Cheap and compact light emitting diodes (LEDs) have become the mainstream light sources for skin PDT with a wide wavelength range, adjustable power output and large irradiation areas. Two-photon PDT using femtosecond solid-state lasers allows spatially precise activation of photosensitisers in tissues with for example 350 nm UVA using more deeply penetrating 700 nm red-light pulses. This may be clinically useful if methods can be developed for fast rastering of the very small (ca. 1 µm) laser spot so that reasonable tumour volumes can be treated in a short time.

Drug administration is another practical issue for clinical PDT.^1,3^ Intravenous injection is the traditional systemic administration for Photofrin^®^ and other tetrapyrrolic photosensitisers. Although it is convenient and efficient for internal organ PDT, high tumour-specific accumulation of photosensitisers is required to improve efficacy and reduce systemic cytotoxicity. By contrast, 5-aminolevulinic acid can be applied topically and orally. For treatment of large areas topically, drugs should have low photocytotoxicity to normal tissue. TLD-1433 is intravesically instilled and relies on high selectivity for bladder tumours over normal tissues.

Despite the wide clinical applications of PDT, it suffers from several problems. Firstly, the mechanism of action is highly dependent on oxygen, which limits the efficacy of PDT in hypoxic tumours. However, oxygen-independent photosensitisers are now becoming available (Fig. 1). For example, a highly oxidative organo-Ir(III) photosensitiser can catalyse the photoreduction of cytochrome *c* synergistically with NADH oxidation under hypoxia, and is phototoxic towards both normoxic and hypoxic cancer cells.^6^ In another strategy, a chlorin photosensitiser has been combined with an oxygen-generating diazido Pt(IV) complex (Ce6-PEG-Pt(IV) conjugate) in upconverting nanoparticles that exhibit dramatically enhanced photocytotoxicity in hypoxic tumour models with NIR (980 nm) irradiation.^7^ Diazido Pt(IV) complexes themselves show potential for a novel form of photoactivated chemotherapy (PACT). They are highly stable in the dark but decompose upon irradiation with visible light to release cytotoxic azidyl radicals and reactive oxygen species, as well as DNA-binding Pt(II) species.^8^ Axial derivatisation of diazido Pt(IV) complexes with anticancer drugs or cancer-targeting vectors can improve the photo-selectivity of the agents and red-shift their activation wavelength.

Cancer-targeting proteins can assist the delivery of photosensitisers to tumours and increase selectivity versus normal tissue. For example, Rutherrin, a conjugate of TLD-1433 and apo-transferrin, exhibits enhanced cancer-targeting and photocytotoxicity.^3^ Due to the abnormal mitochondrial function of cancer cells, mitochondrial-targeting can also lead to improved cancer selectivity. Triphenylphosphonium (TPP) and dichloroacetate (DCA) have been conjugated to photosensitisers as mitochondrial-targeting tags. TPP and a Ru(II) photosensitiser bound to human serum albumin (HSA) as a nanocarrier to form cHSA-PEO-TPP-Ru, exhibits mitochondrial localisation, enhanced ^1^O_2_ generation, cancer accumulation and photocytotoxicity.^9^

Finally, PDT can not treat advanced disseminated disease due to the difficulty of light delivery and the limited penetration depth. Photothermal therapy (PTT) is a new treatment for advanced tumours, in which photosensitisers absorb NIR and release vibrational energy (heat) to kill cancer cells, independent of oxygen.^10^ However, the composition of current PTT photosensitisers, mainly inorganic nanomaterials, needs to be optimised if they are to become clinical drugs.

The introduction of light into cancer treatment can provide precise tumour localisation of treatment and low invasiveness. Due to the novel mechanism of action, phototherapy is not usually cross-resistant with other cancer treatments, and thus can be a component of combination treatments. Current new developments in the design of both light sources and photoactive agents suggest that oncological phototherapy will have wider applications in the future.

## Data Availability

Not applicable.
